# Spontaneous Closure of Full-Thickness Macular Hole in a Patient with Retinal Arteriovenous Malformation: Sixteen-Year Follow-up

**DOI:** 10.1055/a-2550-0839

**Published:** 2025-06-16

**Authors:** Zeynep Serikoglu Akbas, Faik Gelisken, Benedikt Rössler

**Affiliations:** 1 Department of Ophthalmology, Istanbul University Cerrahpasa, Faculty of Medicine, Fatih, Turkey; 2 Department of Ophthalmology, University Hospital Tübingen, Germany

## Introduction

Retinal arteriovenous malformation (RAM), also known as retinal racemose haemangioma (RRH), is a congenital, non-hereditary, and sporadic vascular anomaly characterised by the appearance of dilated and tortuous abnormal retinal vessels. When accompanied by vascular malformations in the midbrain, mandible, or other parts of the body, it is referred to as Wyburn-Masonʼs disease 1 , 2 , 3 .

Although RAM is typically asymptomatic and stable, complications such as central retinal vein occlusion, retinal ischaemia, and vitreous haemorrhage have been documented 4 .

This report details a case of RAM complicated by central retinal vein occlusion and secondary full-thickness macular hole (FTMH) with spontaneous closure during follow-up of sixteen years.

## Case Report

A 20-year-old female was referred to the ophthalmology department of the university hospital presenting with visual loss in the right eye for the previous four weeks. She reported no pain, redness, or photophobia. Her medical history included anaemia diagnosed four months ago. Family history was unremarkable.

**Fig. 1:**
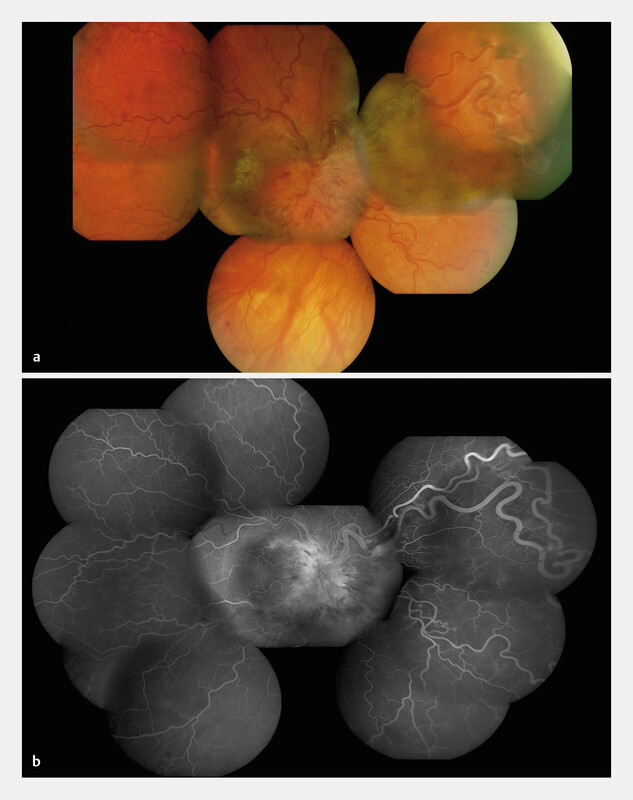
**a** Panoramic colour fundus image of the right eye at first presentation shows papilledema, parapapillary haemorrhages, tortuosity of retinal veins, macular oedema, star-shaped lipid exudates in the fovea and punctate haemorrhages in the periphery consistent with central retinal vein occlusion. In the nasal periphery, a retinal arteriovenous malformation complex is seen. **b** Fundus fluorescein angiography of the right eye at first presentation shows peripapillary hyperfluorescence and an abnormally dilated tortuous retinal vascular complex in the nasal midperiphery.

Best corrected visual acuity (BVCA) was 20/100 in the right eye and 20/20 in the left eye. Intraocular pressure and anterior segment examination of both eyes were normal. In the right eye, fundoscopy revealed papilledema, parapapillary flame-shaped haemorrhages, tortuosity, and dilation of retinal veins, macular oedema, star-shaped lipid exudation in the fovea and peripheral dot-blot haemorrhages. A vascular anomalous complex was seen in the nasal periphery. Fundus was observed to be normal in the left eye. Fundus fluorescein angiography of the right eye demonstrated hyperfluorescence in the peripapillary region and an abnormally dilated tortuous retinal vascular complex in the superonasal midperiphery ( Fig. 1 a and b ).

Dermatological, internal, and neurological investigations including magnetic resonance imaging of the cranium and the orbital cavity did not reveal any abnormalities. The patient was diagnosed with central retinal vein occlusion and unilateral congenital RAM of the right eye.

**Fig. 2:**
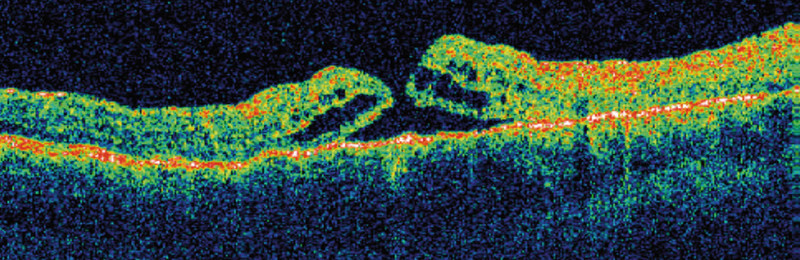
Horizontal OCT scan through the fovea of the right eye four months after the initial presentation shows FTHM and large serous detachment of the macula.

**Fig. 3:**
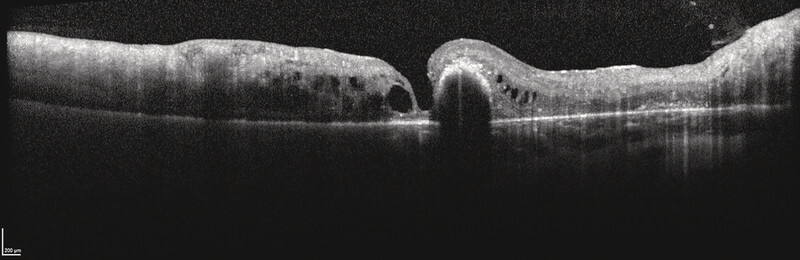
Horizontal OCT scan through the fovea of the right eye nearly four years after the first presentation shows a lamellar macular hole, parafoveal intraretinal cystoid changes and an intraretinal hyperreflective structure nasal to the fovea.

**Fig. 4:**
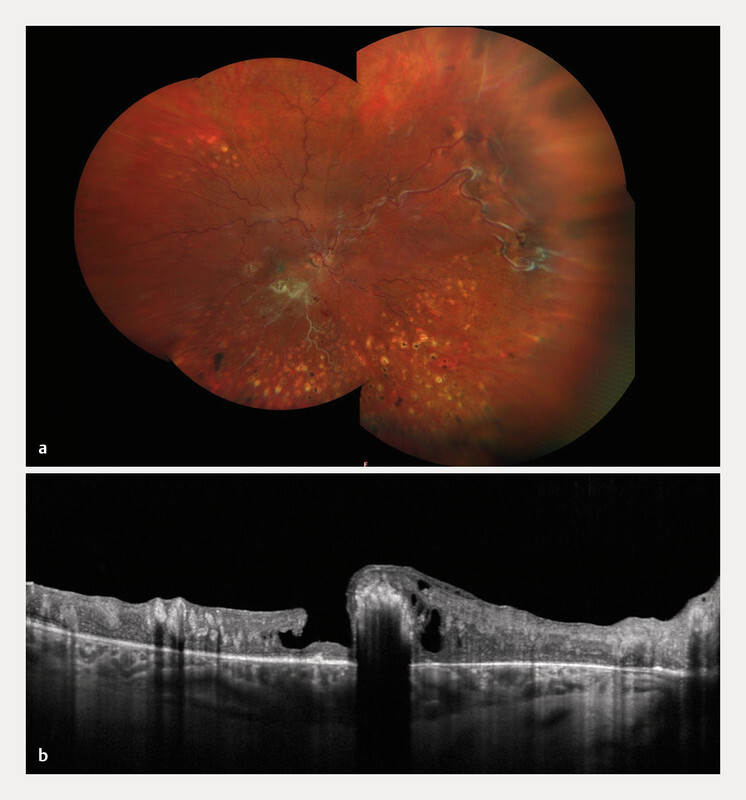
**a** Panoramic colour fundus image of the right eye at the final examination shows papillary venous collaterals, tortuosity and dilation of the retinal veins, exudation in the macula, ghost vessels in the temporal inferior quadrant, laser scars in the inferior quadrants of the fundus and RAM complex with a similar appearance to that observed on the initial examination. **b** Horizontal OCT scan through the fovea of the right eye at the final examination shows a persistent lamellar macular hole, a parafoveal intraretinal hyperreflective structure, intraretinal cystoid cavities, and an epiretinal membrane.

Macular oedema regressed following four intravitreal applications of 1,25 mg Bevacizumab. The time-domain optical coherence tomography (OCT) examination four months after the first presentation revealed a full-thickness macular hole (FTMH) with intra- and subretinal fluid at the macula ( Fig. 2 ). The BCVA was decreased to 20/200. The patient was explicitly informed about the therapeutic option of a macular surgery to treat the FTMH, but denied the surgery. Following the first phase of care in our clinic, the patient gave birth to a child and decided to be cared for by the ophthalmologist close to home for a period of 3 years. Within this period, she received a sectorial laser photocoagulation. In the next follow-up visit three years later, the OCT examination of the right eye revealed a spontaneous closure of the FTMH with a lamellar macular hole formation and an epiretinal membrane ( Fig. 3 ). At the final examination, 16 years after the initial presentation, the right eye exhibited macular exudation, papillary collaterals, retinal vein tortuosity, ghost vessels in the temporal inferior quadrant, laser scars in the inferior quadrants of the fundus and RAM complex with a similar appearance to that observed on the initial examination. The OCT examination revealed a lamellar macular hole, intraretinal cystoid changes, and an ERM ( Fig. 4 a and b ). Her BCVA improved slightly to 20/100.

## Discussion

Retinal arteriovenous malformation is a rare entity. While the majority of cases are asymptomatic, it can potentially lead to visual loss when complications such as retinal vein occlusion occur. The formation of a macular hole in a RAM case was observed once by Muñoz et al. (1991) without any change at six-year follow-up 5 .

The present case had some atypical clinical features. Even though there was clinical visible macular oedema, the late phase of the fluorescein angiography did not show any apparent leakage in the macula. Therefore, a coexistence of a temporary retinal arterial occlusion with reperfusion cannot be excluded, which could have contributed to the macular thickening by intracellular oedema.

It has been reported that macular oedema and intravitreal pharmacotherapy may be involved in the formation of a FTMH. In such eyes, rapid reduction of oedema after intravitreal pharmacotherapy, foveal ischaemia, and cystoid degenerations have been considered as a contributing factor in the pathogenesis of the FTMH 6 , 7 .

In this report, we have described a case of RAM presenting with central retinal vein occlusion and with FTMH development, which closed spontaneously during the follow-up. No vitreous traction was observed. Spontaneous FTMH closure has been documented in various studies. The presence of parafoveal cystoid changes has been identified as a positive predictive feature for hole closure. In such cases, if medical treatment is still ongoing, it is advised to consider a follow-up period before deciding a surgical intervention 8 .

FTMH is a very rare complication in cases of RAM. To the best of our knowledge, this case report is the first presentation of a spontaneously closed FTMH in a RAM patient with long-term follow-up of sixteen years.
